# Optimization of polymyxin B regimens for the treatment of carbapenem-resistant organism nosocomial pneumonia: a real-world prospective study

**DOI:** 10.1186/s13054-023-04448-z

**Published:** 2023-04-28

**Authors:** Tiantian Tang, Ying Li, Ping Xu, Yanjun Zhong, Min Yang, Wanjun Ma, Daxiong Xiang, Bikui Zhang, Yangang Zhou

**Affiliations:** 1 Department of Pharmacy, The Second Xiangya Hospital, Central South University, Changsha, China; 2 Institute of Clinical Pharmacy, Central South University, Changsha, China; 3 Hunan Provincial Engineering Research Centre of Translational Medicine and Innovative Drug, Changsha, China; 4 Department of Critical Care Medicine, The Second Xiangya Hospital, Central South University, Changsha, China; 5 Department of Respiratory and Critical Care Medicine, The Second Xiangya Hospital, Central South University, Changsha, China; 6 Department of Pharmacy, Union Hospital, Tongji Medical College, Huazhong University of Science and Technology, Wuhan, China

**Keywords:** Polymyxin B, Carbapenem-resistant organism, Nosocomial pneumonia, Dosing optimization, Pharmacokinetic/pharmacodynamic

## Abstract

**Background:**

Polymyxin B is the first-line therapy for Carbapenem-resistant organism (CRO) nosocomial pneumonia. However, clinical data for its pharmacokinetic/pharmacodynamic (PK/PD) relationship are limited. This study aimed to investigate the relationship between polymyxin B exposure and efficacy for the treatment of CRO pneumonia in critically ill patients, and to optimize the individual dosing regimens.

**Methods:**

Patients treated with polymyxin B for CRO pneumonia were enrolled. Blood samples were assayed using a validated high-performance liquid chromatography-tandem mass spectrometry method. Population PK analysis and Monte Carlo simulation were performed using Phoenix NLME software. Logistic regression analyses and receiver operating characteristic (ROC) curve were employed to identify the significant predictors and PK/PD indices of polymyxin B efficacy.

**Results:**

A total of 105 patients were included, and the population PK model was developed based on 295 plasma concentrations. AUC_ss,24 h_/MIC (AOR = 0.97, 95% CI 0.95–0.99, *p* = 0.009), daily dose (AOR = 0.98, 95% CI 0.97–0.99, *p* = 0.028), and combination of inhaled polymyxin B (AOR = 0.32, 95% CI 0.11–0.94, *p* = 0.039) were independent risk factors for polymyxin B efficacy. ROC curve showed that AUC_ss,24 h_/MIC is the most predictive PK/PD index of polymyxin B for the treatment of nosocomial pneumonia caused by CRO, and the optimal cutoff point value was 66.9 in patients receiving combination therapy with another antimicrobial. Model-based simulation suggests that the maintaining daily dose of 75 and 100 mg Q12 h could achieve ≥ 90% PTA of this clinical target at MIC values ≤ 0.5 and 1 mg/L, respectively. For patients unable to achieve the target concentration by intravenous administration, adjunctive inhalation of polymyxin B would be beneficial.

**Conclusions:**

For CRO pneumonia, daily dose of 75 and 100 mg Q12 h was recommended for clinical efficacy. Inhalation of polymyxin B is beneficial for patients who cannot achieve the target concentration by intravenous administration.

**Supplementary Information:**

The online version contains supplementary material available at 10.1186/s13054-023-04448-z.

## Background

Over the last decade, nosocomial pneumonia caused by carbapenem-resistant organism (CRO) infection has become a significant important cause of mortality and morbidity worldwide, especially in critically ill patients [1, 2]. Due to the broad antimicrobial resistance among CRO, there is limited treatment option, and make it an extreme challenge [3, 4].

Polymyxins (polymyxin B and colistin), which were withdrawn from the market due to the high risk of nephrotoxicity and neurotoxicity in the 1970s, have been reused for their high sensitivity against CRO [5, 6]. And because of its more predictable pharmacokinetics and rapid antimicrobial activity, polymyxin B has become a preferred choice over colistin [7, 8]. Unfortunately, due to the early development and a subsequent lack of use in a clinical setting, there is little information about the pharmacokinetic/pharmacodynamic (PK/PD) relationship of polymyxin B against CRO pneumonia, and the optimal dosing remains controversial [9–11].

The latest guidelines recommend an area under the concentration-time curve across 24 h at steady state (AUC_ss,24 h_) of 50–100 mg·h/L for polymyxins to achieve bactericidal activity against an isolate with a MIC of 2 mg/L (the EUCAST and CLSI breakpoints) [7]. However, this PK/PD target was mainly based on the results of limited in vitro and murine thigh infection models, and most evaluated colistin [9, 12]. Although Yang et al. recently confirmed that AUC_ss,24 h_ threshold of 50–100 mg·h/L was a good predictor of polymyxin B clinical response and acute kidney injury (AKI) risk in a retrospective study, it has to be pointed out that this study included patients with different types of infections, and was not focus on pneumonia [13]. It is well known that the PK/PD indices and targets of antibiotics are diversity among different types of infections [14, 15]. Moreover, according to the PK/PD analysis of murine lung infection model, the present recommended target is very likely to be suboptimal for the systemic treatment of pneumonia [16]. Therefore, it is necessary to re-evaluate whether this relationship applies to patients with CRO nosocomial pneumonia in prospective clinical trials.

At present, weight-based dosing regimen (1.25–1.5 mg/kg every 12 h) is recommended for polymyxin B [17, 18]. However, polymyxin B concentration varies widely in critically ill patients with this regimen, and almost 30% patients cannot achieve AUC_ss,24 h_ values within the target therapeutic window [19]. Moreover, Miglis et al. found that weight-based dosing strategies might be associated with increased toxicity in higher weight patients as well as insufficient concentration in lower weight patients [20]. Due to these inconsistent results, further research is needed to improve the characterization of polymyxin B PK, in order to identify the optimization of dose regimens.

The primary objective of this study was to investigate the relationship between polymyxin B exposure and efficacy in the treatment of CRO pneumonia and to determine the appropriate PK/PD target for this infection. In addition, Monte Carlo simulations were performed to select the optimal dosage regimens.

## Methods

### Study design and patients

This prospective study was conducted at two intensive care units (ICU) between January 2020 and December 2021 in the Second Xiangya Hospital of Central South University (Changsha, China). Patients were included if (a) age ≥ 18 years; (b) diagnosed with nosocomial pneumonia that developed more than 48 h after admission; (c) at least two consecutive samples on different days (time interval at least 24 h) showed the presence of CRO from bronchial secretions or bronchoalveolar lavage samples; (d) received intravenous polymyxin B treatment over 3 days. The exclusion criteria were as follows: (a) concomitant lung cancer with obstructive pneumonitis or cystic fibrosis; (b) solid organ transplantation; (c) hematologic malignancies and hematopoietic cell transplant recipients; (d) receiving renal replacement therapy. HAP was defined according to the 2016 clinical practice guidelines of the Infectious Diseases Society of America and the American Thoracic Society [21]. Determination of carbapenem susceptibility of CRO was followed by the European Committee on Antimicrobial Susceptibility Testing (EUCAST). Updated EUCAST Clinical Breakpoints of polymyxin B were sensitivity (S ≤ 2 mg/L) and drug resistance (R > 2 mg/L). The antimicrobial susceptibility testing was performed using the VITEK-2 Compact system with VITEK cards (0.5–16 mg/L for colistin) (bioMérieux, France). The following information was extracted from the electronic medical records: demographic and co-morbidity profiles, clinical and microbiological features of the infections, and the antimicrobial treatment regimens. Creatinine clearance (CrCL) was calculated using the Cockcroft–Gault equation. The endpoint was clinical efficacy and 30-day all-cause mortality. Assessment of clinical efficacy was conducted at the end of treatment, and 30-day mortality was recorded from the start of polymyxin B treatment. This prospective study was approved by the Ethics Committee of the Second Xiangya Hospital, Central South University. Informed consent was obtained from all patients or legal representatives of the patients (No. ChiCTR1900022231).

### Drug administration and concentration determination

Polymyxin B was given to all patients empirically as a loading dose of 100–200 mg followed by a maintenance dose of 40–100 mg every 12 h for at least 3 days. The infusion time was at least 1 h. Aerosol delivery of polymyxin B (25 mg or 50 mg twice daily) was using a vibrating mesh nebulizer, synchronized with the inspiratory cycle of the ventilator. Two to six blood samples (2 mL) were randomly collected immediately before the seventh dose of polymyxin B and at 0, 1, 2, 4, 6, 8 and 10 h after the end of infusion. The supernatant was immediately stored at − 80 °C until analysis.

An established high-performance liquid chromatography-tandem mass spectrometry (HPLC–MS/MS) was used to measure the concentrations of polymyxin B_1_ and polymyxin B_2_ as described previously by the authors’ laboratory (The total concentration of polymyxin B = [polymyxin B_1_ concentration/polymyxin B_1_ molecular + polymyxin B_2_ concentration/polymyxin B_2_ molecular]*total polymyxin B molecular) [22]. The interday precision was < 12%, the intraday precision was < 9%, and the accuracy ranged from 96.1 to 110.4%. The limit of quantification (LLOD) was 0.03 mg/L, and all of the polymyxin B concentrations detected were over LLOD.

### Population PK model and calculation of PK/PD indices

The Phoenix NLME program (version 8.1. Pharsight, A Certara Company, USA) with the method of first-order conditional estimation-extended least square method (FOCE-ELS) was used to develop the population PK model by analyzing polymyxin B concentration. The objective function value (OFV), goodness-of-fit plots and the reasonable of population PK parameters were used to selection of the structure model. The stepwise covariate modeling (SCM) approach was used to test the covariate model in this analysis; age, sex, body weight, alanine aminotransferase (ALT), aspartate aminotransferase (AST), total bilirubin (TBIL), direct bilirubin (DBIL), serum albumin (ALB) and CrCL were evaluated as the covariates. The SCM consists of a forward selection step (the criterion is *p* < 0.05 for ΔOFV decreased ≥ 3.84) and a backward elimination step (the criterion is *p* < 0.001 for ΔOFV increased ≥ 10.83). In addition, the goodness-of-fit plots were used to assess the validity of the population PK model, the prediction-corrected visual predictive check (pcVPC) was used to assess the predictive performance of the key models. The bootstrap method was used to assess the accuracy.

The PK/PD indices included AUC_ss,24 h_, AUC_ss,24 h_/MIC, the peak and trough concentration at steady state (*C*_max,ss_ and *C*_min,ss_), *C*_max,ss_ /MIC and *C*_min,ss_ /MIC. AUC_ss,24 h,_ *C*_max,ss_ and *C*_min,ss_ were calculated based on the empirical Bayesian (EBEs). For those patients with more than one baseline pathogen, AUC_ss,24 h_/MIC, *C*_max,ss_ /MIC and *C*_min,ss_ /MIC evaluations were based on the pathogen with the highest MIC value.

### Pharmacokinetic/Pharmacodynamic analysis for clinical efficacy and mortality

Clinical outcomes were classified as clinical success (CS) and clinical failure (CF), and were assessed by two physicians. CS was defined as a composite of survival; hemodynamic stability; body temperature < 38 °C, improved biochemistry indicators of infection; stable or improved PaO_2_/FiO_2_ ratio. Additionally, for patients with bacteremia, microbiological cure (no growth of the initial isolate in blood cultures) must be achieved by the end of the treatment [23–25]. Patients who did not meet all above criteria were classified as CF. 30-day all-cause mortality was recorded from the start of polymyxin B treatment.

Variables potentially related to clinical efficacy and 30-day all-cause mortality were assessed, including: demographics, co-morbidities, clinical conditions, dosage regimen and concentration of polymyxin B. To develop receiver operating characteristic (ROC) curves, the PK/PD indices AUC_ss,24 h_/MIC, *C*_max,ss_/MIC and *C*_min,ss_/MIC were used as predictors of clinical efficacy. The area under the diagnostic curve (AUC_ROC_) was calculated to evaluate the correlation of the above parameters with clinical efficacy and 30-day all-cause mortality. Youden index of the ROC curves was calculated by "sensitivity + specificity-1", and the values corresponding to the maximum Youden index is the optimal cutoff point value of the PK/PD indices.

### Monte Carlo simulations of dosage regimens

Based on the final population PK models, the plasma concentration-time profile of 1,000 individuals was simulated. The dosages were selected according to the most commonly used regimens in clinical practice. The regimens were 100–200 mg loading dose followed by 75–150 mg every 12 h. The infusion time was set to 2 h.

### Statistical analysis

Statistical analysis was performed with SPSS 24.0 (SPSS, IBM Company, Chicago, IL, USA) software. Continuous variables are presented as the mean ± standard deviation (SD) if normally distributed and were compared using Student’s *t* tests. The median and interquartile range (IQR) are presented for abnormally distributed data, and the Mann–Whitney *U* test was used. Categorical variables are expressed as counts and percentages, and the chi-square test or Fisher’s exact test was used. Spearman’s rank correlation coefficient (r) was used to analyze the correlation between *C*_min,ss_, *C*_max,ss_ and AUC_ss,24 h_. Univariate analysis was performed for all variables to identify possible predictors for clinical efficacy. Variables with a *p* < 0.05 were entered into the multivariate logistic regression models. A forward stepwise (likelihood ratio) method was performed to determine the predictors using a significance level of 0.05 for entry and 0.10 for removal from the model. 30-day all-cause mortality was evaluated with Cox regression model. *P* < 0.05 was considered statistically significant.

## Results

### Patients characteristics

During the study period, 132 patients (≥ 18 years) received polymyxin B therapy. Among them, 11 patients were non-HAP, five patients had no pathogenic microorganism result, four patients were solid transplant recipients, four patients received renal replacement and three patients received polymyxin B treatment ≤ 3 days. Thus, 105 patients were eventually enrolled. The demographic characteristics of all patients are summarized in Table 1. The median APACHE II score of these patients was 18 (IQR: 12, 25), and the rate of sepsis was 39.0% (41/105). The most common pathogenic bacteria were *Acinetobacter baumannii* (*N* = 86; 81.9%), followed by *Klebsiella pneumoniae* (*N* = 42; 40.0%) and *Pseudomonas aeruginosa* (*N* = 14; 13.3%). For most of our CRO stains, MIC values of polymyxin B were 1 mg/L, and ≤ 0.5 mg/L for three isolates of *Klebsiella pneumoniae*. MIC_50_ and MIC_90_ of polymyxin B were 1 mg/L for *Acinetobacter baumannii*, *Klebsiella pneumoniae and Pseudomonas aeruginosa*. The loading dose of polymyxin B was 100 mg (IQR: 100,150 mg). The median polymyxin B daily dose was 2.3 mg/kg (IQR: 2.0, 2.9 mg/kg) with a duration of 12 days (IQR: 9, 16).

**Table 1:** Demographic data for 105 patients in PPK model

Variable	Values^a^
*Demographics*	
Age (years)	65 (IQR: 55, 76)
Gender (male)	76 (72.4%)
Weight (Kg)	55.0 (IQR:50.0, 65.0)
*Clinical condition*	
Albumin (g/L)	30.7 (IQR: 27.9, 34.2)
Baseline creatinine clearance (mL/min)	72.2 (IQR: 50.5, 111.5)
Baseline BUN (mmol/L)	9.8 (IQR: 6.6, 15.8)
Mechanical ventilation	81 (77.1%)
APACHEII scores	18 (IQR: 12, 25)
Mortality rates	28 (26.7%)
*Comorbidities*	
Sepsis	41 (39.0%)
Pulmonary diseases	15 (14.3%)
Heart disease	58 (55.2%)
Diabetes mellitus	19 (18.1%)
Chronic liver disease	29 (27.6%)
Chronic renal dysfunction	25 (23.8%)
Trauma	5 (4.8%)
Solid tumor	15 (14.3%)
*Pathogens*	
CRAB	86 (81.9%)
CRKP	42 (40.0%)
CRPA	23 (21.9%)
*PMB treatment*	
PMB loading dose (mg) (*n* = 87)	100 (IQR: 100, 150)
PMB loading dose (mg/kg) (*n* = 87)	2.1 (IQR: 1.9, 2.3)
PMB daily dose (mg)	150 (IQR: 100, 150)
PMB daily dose by weight (mg/kg/day)	2.3 (IQR: 2.0, 2.9)
PMB treatment duration (days)	12 (IQR: 9, 16)
PMB total dose (mg)	1500 (IQR: 1100, 2175)
PMB total dose by weight (mg/kg/day)	26.0 (IQR: 18.7, 38.2)
*Combinational therapy*	
Carbepenem	36 (34.3%)
Tigecycline	33 (31.4%)
*Other β-lactam antibiotics*^b^	32 (30.5%)
Ceftazidime avibactam	4 (3.8%)
Quinolone	4 (3.8%)

*IQR* Interquartile range, *APACHE* Acute physiology and chronic health evaluation, *BUN* Blood urea nitrogen. *PMB* Polymyxin B, *CRAB* Carbapenem-resistant *acinetobacter baumannii*, *CRKP* Carbapenem-resistant *klebsiella pneumonia*, *CRPA* Carbapenem-resistant *pseudomonas aeruginosa*

^a^Categorical data are number (%) of subjects, continuous data are expressed as median (interquartile range, IQR)

^b^other β-lactam antibiotics include cefoperazone/sulbactam (*n* = 23) and piperacillin/tazobactam (*n* = 9)

### Population PK Model and polymyxin B exposure

The population PK model was developed based on 295 plasma concentrations obtained from 105 patients, each patient on average contributed three clinical samples. A two-compartment model fully described the data, and no covariate was statistically significant to PK parameters. A proportional error model was used to evaluate the residual variability. In addition, the shrinkage of the clearance between central compartment and peripheral compartment (CLd) and volume in peripheral compartment (Vp) were more than 50%, so the inter-individual variability (IIV) of CLd and Vp were fixed 0. Due to the highly correlation between the clearance in central compartment (CL) and the volume distribution in central compartment (Vc), the omega block was applied to improve the accuracy of IIV in each patient, and the estimated covariance between CL and Vc is 100%.

**Fig. 1:**
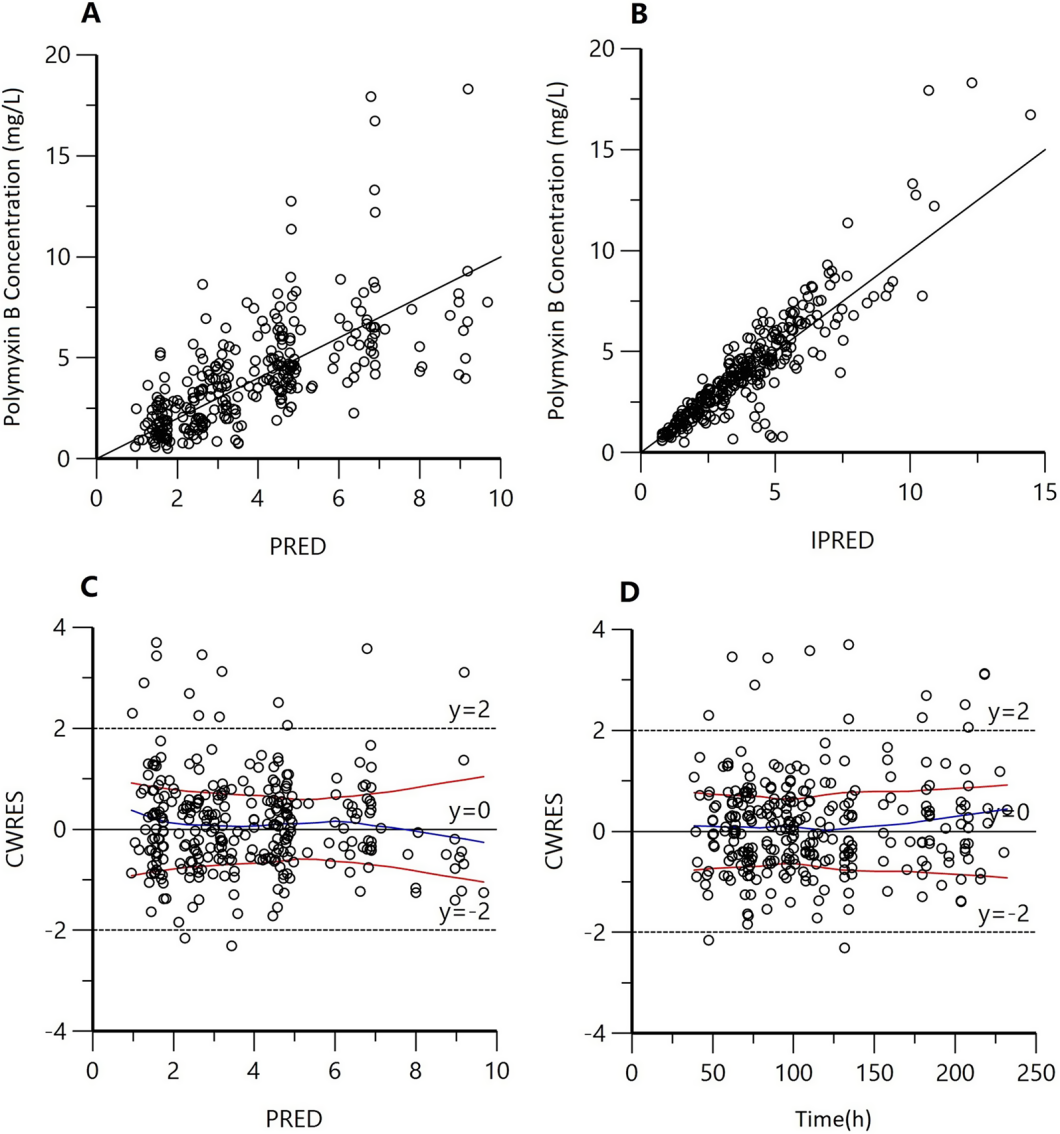
Goodness-of-fit plots for the final population pharmacokinetic model. **A** Polymyxin B concentration versus population predicted concentrations (PRED); **B** Polymyxin B concentration versus individual predicted concentrations (IPRED); **C** Conditional weighted residuals versus population predicted concentrations (CWRES vs. PRED); **D** Conditional weighted residuals versus time (CWRES vs. Time); The blue lines in panels (**C**, **D**) represent smoothed regression lines

**Fig. 2:**
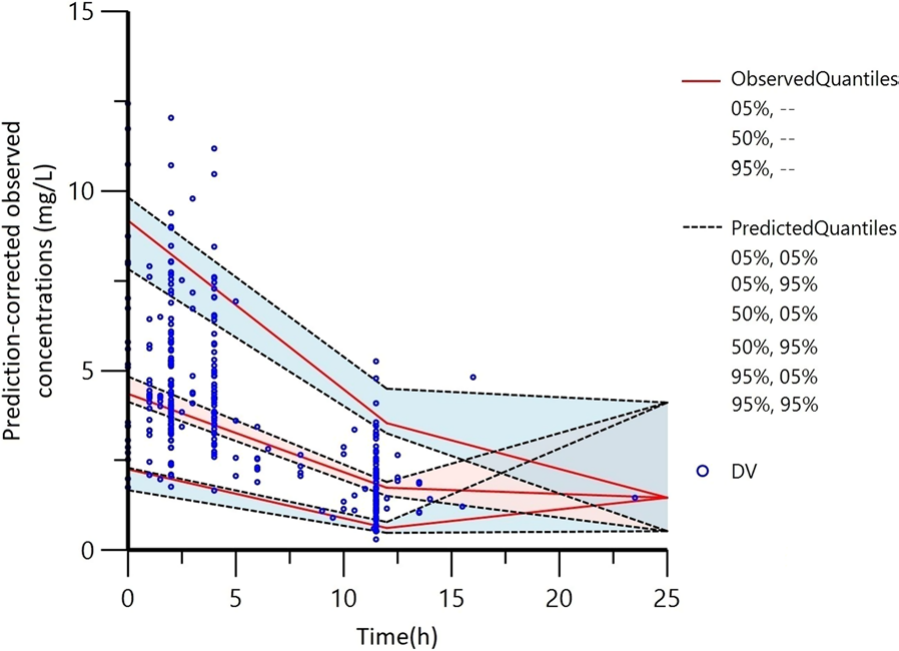
The prediction corrected-visual predictive check (pc-VPC) of the final population PK model. The red and black lines represent the 5th, 50th and 95th quantiles of the observed and predicted concentration, and the shaded area represents the simulation-based 90% confidence intervals

The goodness-of-fit plots in the final model are shown in Fig. 1. The plots were shown that the structure of the final model was not biased and that the model was acceptable. The result of pcVPC is presented in Fig. 2. Most concentrations were within the 90% CIs, indicating that the final model had a good description of the original data. The bootstrap results are shown in Table 2, which indicating qualified precision for the final population PK models. In the final model, the typical values of CL, Vc, CLd, and Vp were 1.56 L/h, 12.5 L, 2.41 L/h, and 29.9 L, respectively. The final PK model equations were as follows: $\mathrm{CL}\left(L/h\right)=1.56*\mathrm{exp}(\eta \mathrm{CL})$, $\mathrm{Vc}\left(L\right)=12.5*\mathrm{exp}(\eta \mathrm{Vc})$, $\mathrm{CLd}\left(L/h\right)=2.41$, $\mathrm{Vp}(L)=29.9$. The results of final PPK model are shown in Table 2.

**Table 2:** Population PK parameter estimates in the final model and bootstrap

Final model			Bootstrap method		
Parameters (Unite)	Estimate	Standard Error (%SE)	Parameters (Unite)	Estimate	Standard Error (%SE)
Fixed effect			Fixed effect		
CL (L/h)	1.56	4.50	CL (L/h)	1.56	4.47
CLd (L/h)	2.41	11.8	CLd (L/h)	2.41	11.6
Vc (L)	12.5	5.67	Vc (L)	12.5	5.65
Vp (L)	29.9	17.3	Vp (L)	29.9	16.7
Random effect (%)			Random effect (%)		
ω_CL*Vc_	100	12.7	*ω*_CL*Vc_	100	12.5
ω_CL_	39.4	6.4	*ω*_CL_	39.4	6.16
ω_Vc_	28.6	15.5	*ω*_Vc_	28.6	15.3
Residual error (%)			Residual error (%)		
Proportional residual (%)	27.86	9.11	Proportional residual (%)	27.86	9.09

*CL* The clearance in central compartment, *CLd* The clearance between central compartment and peripheral compartment, *Vc* The volume distribution in central compartment, *Vp* The volume in peripheral compartment, *ω*_CL*Vc_ The correlation of CL and Vc

The median values of *C*_min,ss_, *C*_max,ss_ and AUC_ss,24 h_ were 1.9 mg/L (IQR:1.4, 2.7 mg/L), 5.9 mg/L (4.6, 7.1 mg/L) and 67.2 mg·h/L (IQR:54.8, 84.2 mg·h/L), respectively. Spearman’s rank correlation analysis showed that AUC_ss,24 h_ were positively correlated *C*_min,ss_, *C*_max,ss_ (Additional file 1: Fig. S1a, Additional file 2: Fig. S1b).

### Pharmacokinetic/Pharmacodynamic analysis for clinical efficacy and mortality

**Fig. 3:**
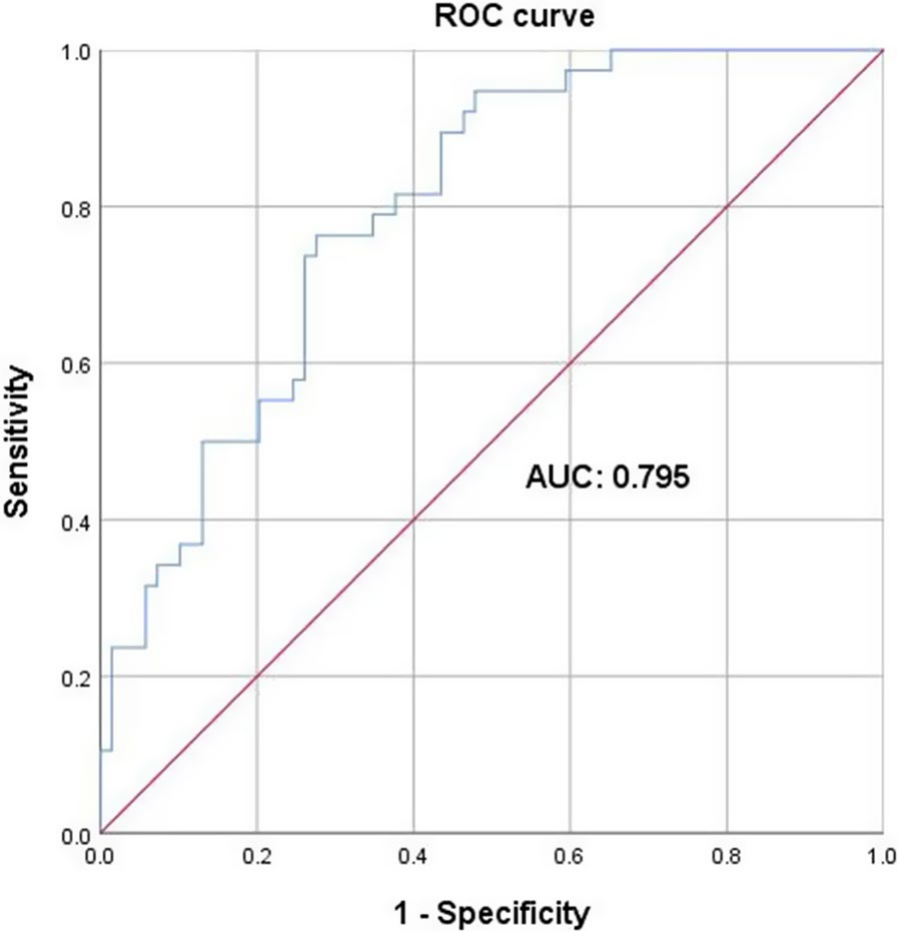
ROC of the final multivariate logistic regression model

Among the 105 patients, the clinical success rate was 66.7% (70/105). Patients in the clinical success group were more likely to combine with inhaled polymyxin B (40.0% VS 17.1%, *p* = 0.012) than patients in the clinical failure group. The dosage (daily, total), duration and plasma concentrations (*C*_min,ss_, *C*_max,ss_, AUC_ss,24 h_, *C*_min,ss_/MIC, *C*_max,ss_/MIC and AUC_ss,24 h_/MIC) of polymyxin B were significantly higher (*p* < 0.05) in the clinical success group than in the clinical failure group (Table 3). Based on these results, a total of 12 possible risk factors were identified by the univariate analysis. These 12 variables (*p* < 0.05) were used to develop a multivariate logistic regression model. Finally, AUC_ss,24 h_/MIC (AOR = 0.97, 95% CI 0.95–0.99, *p* = 0.009), daily dose (AOR = 0.98, 95% CI 0.97–0.99, *p* = 0.028), and combination of inhaled polymyxin B (AOR = 0.32, 95% CI 0.11–0.94, *p* = 0.039) were included in the final model. A ROC curve was used to calculate the discriminatory power of the model (AUC = 0.795) (Fig. 3). In addition, considering that AUC_ss,24 h_ and dose are directly correlated, the relationship between efficacy and dose normalized-AUC_ss,24 h_/MIC was tested using the Covariate-Adjusted Residuals method to remove this intrinsic correlation (AOR = 0.62, 95% CI 0.38–1.01, *p* = 0.056).

**Table 3:** Univariate and Multivariable logistic regression model for clinical efficacy

Variables	CS^a^ (n = 70)	CF^a^ (n = 35)	*p*^b^	Adjusted OR (95% CI)	*p*^c^
*Demographic parameters*					
Female	19 (27.1%)	13 (37.1%)	0.443		
Age (years)	65.4 (56.0, 73.0)	62.2 (51.0, 79.0)	0.259		
Weight (Kg)	59.5 (50.0, 65.0)	54.0 (50.0, 60.0)	0.109		
*Comorbidities*					
Sepsis	22 (31.4%)	19 (54.3%)	0.060		
Pulmonary diseases	12 (17.1%)	3 (8.6%)	0.105		
Heart disease	40 (57.1%)	18 (51.4%)	0.332		
Diabetes mellitus	12 (17.1%)	7 (20.0%)	0.868		
Chronic liver disease	17 (24.3%)	12 (34.3%)	0.415		
Chronic renal dysfunction	13 (18.6%)	12 (34.3%)	0.130		
Solid tumor	10 (14.3%)	5 (14.3%)	0.871		
Clinical conditions					
Baseline CrCL (mL/min)	90.3 (57.5, 102.1)	73.0 (29.3, 104.9)	0.092		
Albumin (g/L)	30.6 (27.9, 32.4)	32.0 (27.0, 35.9)	0.326		
Baseline BUN (mmol/L)	11.7 (6.2, 12.0)	14.0 (7.3,18.2)	0.246		
APACHEII score	17.5 (12, 19)	20.2 (12,25)	0.091		
Mechanical ventilation	49 (62.8%)	32 (91.4%)	0.109		
*Pathogens and susceptibility*					
CRAB	56 (80.0%)	30 (85.7%)	0.897		
CRKP	27 (38.6%)	15 (42.9%)	0.614		
CRPA	13 (18.6%)	10 (28.6%)	0.52		
≤ 0.5 mg/L	3 (4.3%)	0 (0.0%)	0.999		
1 mg/L	67 (95.7%)	35 (100%)	0.999		
*PMB treatment*					
Daily dose (mg)	144.7 (100.0, 150.0)	117.0 (100.0,150.0)	**< 0.0001**	0.98 (0.97–0.99)	0.028
Daily dose/weight (mg/Kg)	2.5 (2.1, 2.9)	2.0 (1.8, 2.5)	**0.004**		
Duration (day)	13 (9, 16)	11 (6, 14)	**0.034**		
Total dosage (mg)	1915.4 (1200.0, 2250.0)	1284.0 (800.0, 1500.0)	**0.001**		
Total dosage (mg/Kg)	33.3 (22.5, 40.0)	24.0 (15.4, 28.0)	**0.002**		
Combined with inhaled PMB	28 (40.0%)	6 (17.1%)	**0.012**	0.32 (0.11–0.94)	0.039
*PMB concentration*					
AUC_ss, 24 h_ (mg·h/L)	78.6 (62.4, 84.2)	60.6 (43.5, 66.6)	**0.002**		
AUC_ss, 24 h_ /MIC	80.5 (62.6, 86.9)	61.9 (43.2, 68.7)	**0.001** 0.056^**#**^	0.97 (0.95–0.99)	0.009
*C*_min,ss_ (μg/mL)	2.4 (1.6, 2.8)	1.8 (1.0, 2.5)	**0.015**		
*C*_min,ss_ /MIC	2.4 (1.4, 3.1)	1.7 (0.9, 1.9)	**0.011**		
*C*_max,ss,_(μg/mL)	6.8 (5.0, 7.6)	5.1 (3.7, 6.9)	**0.005**		
*C*_max,ss_ /MIC	6.7 (4.9, 7.6)	5.0 (3.8, 6.8)	**0.003**		
*Combination therapy*					
Carbapenems	20 (28.6%)	16 (45.7%)	0.156		
Tigecycline	21 (30.0%)	12 (34.3%)	0.865		
Ceftazidime avibactam	3 (4.3%)	1 (2.9%)	0.667		
Other β-lactam^d^	23 (32.8%)	9 (25.7%)	0.321		
Quinolone	4 (5.7%)	0 (0.0%)	0.999		

*CI* Confidence interval, *OR* Odds ratio, *CrCL* Creatinine clearance, *APACHE* Acute physiology and chronic health evaluation, *BUN* Blood urea nitrogen, *PMB* Polymyxin B, *CRAB* Carbapenem-resistant *acinetobacter baumannii*, *CRKP* Carbapenem-resistant *klebsiella pneumonia*, *CRPA* Carbapenem-resistant *pseudomonas aeruginosa*, *AUC*_*ss, 24 h*_ The area under the plasma concentration–time curve across 24 h at steady state, *C*_*min,ss*_ Steady-state trough plasma concentration, *C*_*max,ss*_ Steady-state peak plasma concentration, *CS* Clinical success, *CF* Clinical failure

^a^Categorical data are number (%) of subjects, continuous data are expressed as median (interquartile range, IQR)

^b^derived from univariate analysis

^c^derived from Cox regression analysis

^d^other β-lactam antibiotics include cefoperazone/sulbactam (*n* = 23) and piperacillin/tazobactam (*n* = 9)

^#^adjusted by daily dose. Bold indicates data with significant differences (*p* < 0.05)

**Fig. 4:**
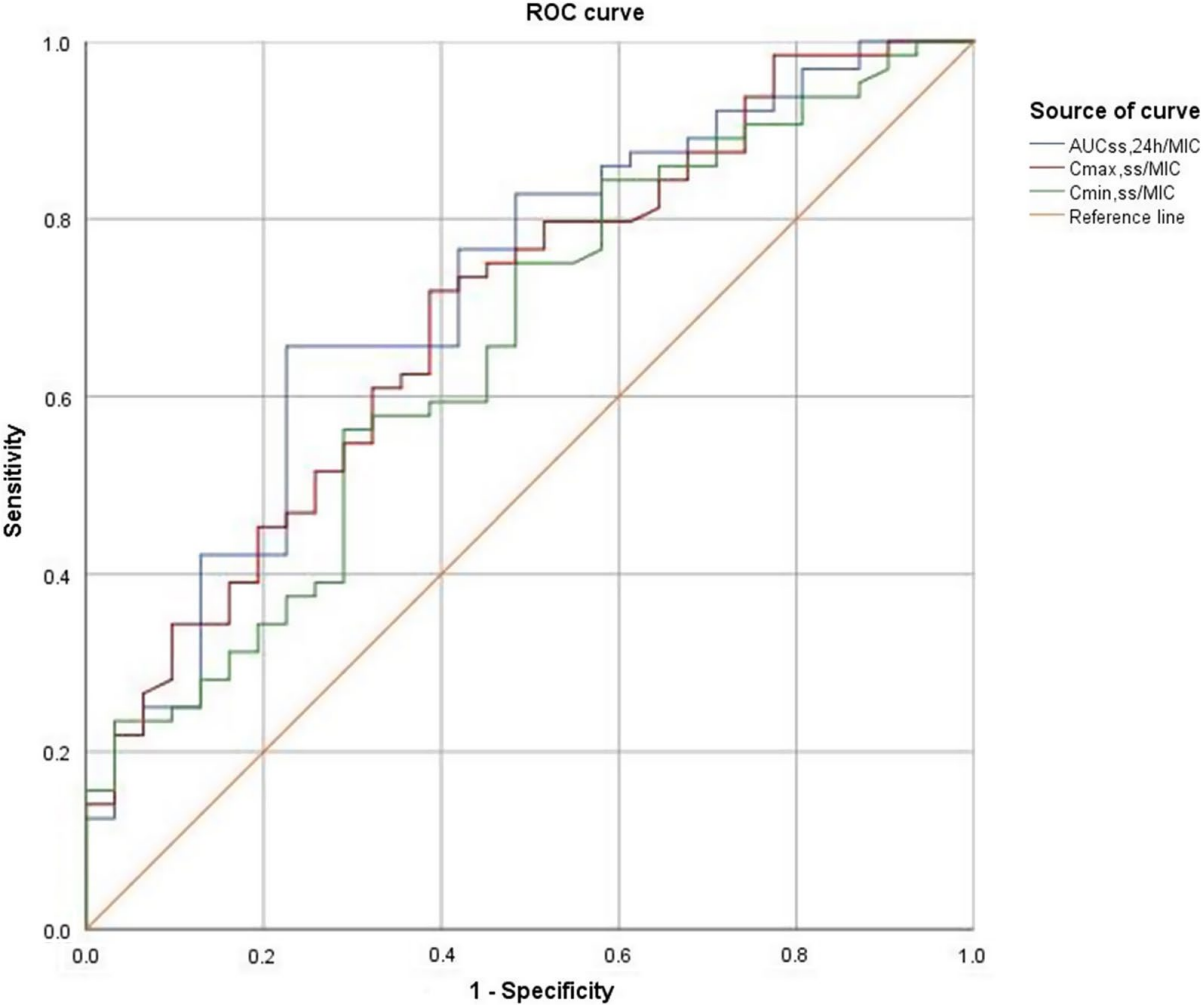
The area under the ROC curve of polymyxin B PK/PD indices in prediction of clinical efficacy

In addition, the ROC curves support that the AUC_ss,24 h_/MIC (AUC_ROC_ = 0.719) was superior to the other two PK/PD indices for the prediction of clinical efficacy. When the Youden index was the largest (1.401), the corresponding optimal cutoff point value of AUC_ss,24 h_/MIC was 66.9. The predictive sensitivity and the specificity of this value were 65.6% and 77.4%, respectively (Fig. 4).

**Fig. 5:**
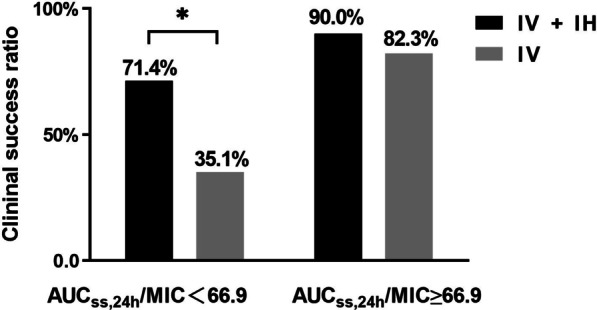
Comparison of clinical efficacy rates of polymyxin B in different subgroups. IV, intravenous polymyxin B; IH, inhaled polymyxin B; * represented *p* < 0.05

Subgroup analysis showed that combination of inhaled polymyxin B was a significant predictor for the clinical efficacy (OR: 0.21, 95% CI: 0.06–0.79, *p* = 0.022) of the 51 patients with AUC_ss,24 h_/MIC < 66.9. The clinical success rate was significantly higher in the intravenous (IV) plus inhaled (IH) group (71.4% vs. 35.1%, *p* = 0.017). However, of the 54 patients with AUC_ss,24 h_/MIC ≥ 66.9, the combination of inhaled polymyxin B was not a significant predictor of the clinical efficacy (OR: 0.46, 95% CI: 0.09–2.46, *p* = 0.365), the clinical success rate in IV + IH group (90.0%) was higher than that in IV group (82.3%), but there was no difference between the two groups (*p* = 0.279) (Fig. 5).

The 30-day all-cause mortality was 26.7% (28/105). None of the investigated PK/PD indices was significantly associated with 30-day mortality, and patients with higher APACHEII score, shorter treatment duration and clinical failure were associated with higher mortality in Cox regression model (Additional file 3: Table S1). ROC analysis was not conducted for mortality since no PK/PD index correlated with it was found in Cox regression model.

### Monte Carlo simulations of dosage regimens

Our PK/PD analysis results showed that AUC_ss,24 h_/MIC over 66.9 was associated with better clinical efficacy. Therefore, this cutoff value was adopted as PK/PD target and the probability of target attainment (PTA) was estimated for seven different MICs ranging from 0.125 to 8 mg/L on day 1 and day 4, respectively.

**Fig. 6:**
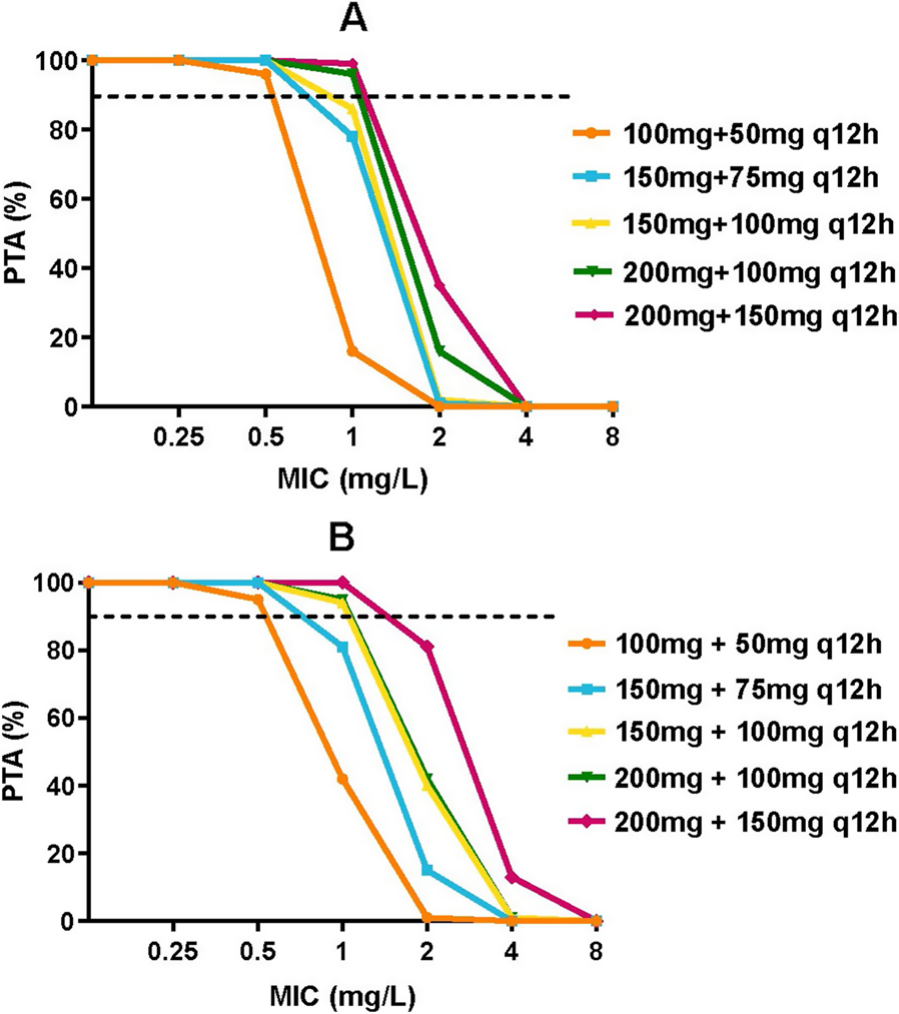
Probability of target attainment (PTA) for polymyxin B regimens on day 1 (**A**) and day 4 (**B**)

For an MIC less than 0.5 mg/L, the PTAs of all simulated regimens were greater than 90% on Day 4; When the MIC value was less than 1 mg/L, regimens from 100 to 150 mg every 12 h achieved the target exposure of AUC_ss,24 h_/MIC over 66.9. None of the simulated regimens achieved adequate target attainment at the current CLSI and EUCAST breakpoint of 2 mg/L. Furthermore, we found that a loading dose made it possible to achieve the target PTA on Day 1, indicating that the loading dose is essential for polymyxin B treatments (Fig. 6).

## Discussion

This study investigated the exposure-response relationship of polymyxin B in the treatment of CRO pneumonia in critically ill patients, as well as explored the optimal dosage regimens for these patients. As a result, AUC_ss,24 h_/MIC is the most predictive PK/PD index of polymyxin B against this infection, with a clinical cutoff value of 66.9. The daily dose of 75 mg and 100 mg Q12 h could achieve ≥ 90% PTA of this clinical target at MIC values ≤ 0.5 and 1 mg/L, respectively.

Several clinical studies have identified that the severity of disease and polymyxin B dosage were significant associating with its efficacy, in according with these results, we found that polymyxin B exposure at the site of infection (daily dose, AUC_ss,24 h_/MIC and combined with inhalation) was the significant predictor of its clinical efficacy in the treatment of CRO pneumonia [26–28].

In *vitro* PD study identified the polymyxins against gram-negative bacteria in a rapid concentration-dependent way, which makes the *f*AUC/MIC and *f*C_max_/MIC the reasonable PK/PD indices [29]. Previous murine thigh and lung infection models identified that *f*AUC/MIC was predictive for the PK/PD index of polymyxins against gram-negative bacteria [16]. Our real-world data further confirmed this finding that AUC_ss,24 h_/MIC is the PK/PD parameter most closely linked to clinical outcomes. In addition, C_max,ss_/MIC also showed good correlation with polymyxin B efficacy (AUC_ROC_ = 0.696; *p* = 0.002), and we found that C_max,ss_ was positively correlated with AUC_ss,24 h_ (Additional file 1: Fig. S1a, Additional file 2: Fig. S1b). In the clinical setting, since obtaining multiple samples throughout a dosing interval to estimate AUC_ss,24 h_ is not always feasible, the limited sampling strategies might be more applicable in clinical practice to assist therapeutic drug monitoring of polymyxin B, further investigation with a large sample may help to confirm this correlation.

According to the results of mouse lung infection models, the present recommended PK/PD target (AUC_ss,24 h_ of 50–100 mg·h/L) is supposed to be suboptimal for the systemic treatment of pneumonia [7, 16]. However, using the ROC curve, we identified the clinical cutoff value of AUC_ss,24 h_/MIC (66.9), as the MIC values of polymyxin B for most of the CRO strains in our study are 1 mg/L, it seems that polymyxin B can lead to favorable clinical outcomes in pneumonia patients with AUC_ss,24 h_ > 66.9 (85.2%). The causes of this inconsistency might be as follows: 1. previous preclinical studies investigated the PK/PD target in the neutropenic murine model, but none of our patients was immunodeficient, and they might response better to the antibiotic treatment; 2. the recommended PK/PD exposure targets were derived from studies involving polymyxins monotherapy, however, all of our patients received combination therapy which is advantageous in the polymyxins treatments. Therefore, the clinical cutoff value of AUC_ss,24 h_/MIC of 66.9 found in our study might be a promising PK/PD target for polymyxin B efficacy in patients receiving combination therapy with another antimicrobial, further study with larger sample is needed to confirm the target.

At present, weight-based dosing regimen is recommended for polymyxin B [17]. However, the relationship between body weight and polymyxin B PK parameters remains controversial [17, 20, 22]. We found no correlation between body weight and polymyxin B PK parameters in this study, which might due to the limited samples of the PPK model and a relatively narrow distribution of patient weights (IQR 55–76 kg), future PPK researches with rich sampling schedules are needed to illuminate the pharmacokinetic characteristics of polymyxin B, and to identify the optimization of dose regimens. According to the results of Monte Carlo simulation, regimens from 100 to 150 mg Q12 h and 75 mg Q12 h could achieve the efficacious target at MIC values ≤ 1 and 0.5 mg/L, respectively. Considering that most of the polymyxin B MIC distributions and MIC_50_/MIC_90_ values for the clinical isolates CRO strains are between 0.5 and 1 mg/L, and polymyxin B daily dose over 200 mg is found to be significantly associated with AKI [26, 30, 31], therefore, a maintenance dose of 75 mg or 100 mg Q12 h might be appropriate.

Adjunctive polymyxin inhale therapy for MDR gram-negative HAP or ventilator associated pneumonia (VAP) is recommended by the guideline [7, 32–34], and in our study, it was also an independent predictor of the clinical efficacy of polymyxin B. Interestingly, additional use of aerosolized polymyxin B did not significantly improve the clinical efficacy in the high exposure (AUC_ss,24 h_/MIC > 66.9) subgroup. However, in the low exposure (AUC_ss,24 h_/MIC < 66.9) subgroup, the use of aerosolized polymyxin B was an independent factor associated with favorable clinical outcome. These results were consistent with the finding by Chen et al. [35], that low-dose intravenous plus inhaled polymyxin B can significantly improve the clinical efficacy of the treatment of VAP. These findings indicated that combining inhalation polymyxin B is especially important for patients with lower exposure. Moreover, with the widespread clinical application of polymyxin B, increased MIC value and resistance have been reported [36–38]. In order to achieve the PK/PD target for these less sensitive bacteria, higher intravenous dosage are required, which may exceed the AKI threshold. Accordingly, combination of inhaled polymyxin B may be a solution to balance the efficacy and toxicity. Therefore, for the treatment of CRO pneumonia, inhaled polymyxin B can not only improve the efficacy, but also avoid the occurrence of AKI.

This study has several limitations. First, the sample size was limited, thus the ability to evaluating the impact of covariates on the population PK parameters is restricted. Second, we did not assess the free concentration of polymyxin B, considering the large protein binding variation among patients [39], the total concentration of polymyxin B might not be in accordance with the unbound fraction, which is considered to be pharmacologically active. To better evaluate the PK/PD relationship of polymyxin B, further study using free drug concentration is needed. Third, polymyxin B MIC values were determined by VITEK 2 automated system in our study, which might lead to onefold to twofold bias of the MIC values, further research using more precise measurement such as broth microdilution (BMD) is needed to clarify our exposure-response results. Last, due to the limited sample size, we cannot compare the efficacy between different inhaled polymyxin B dosages, to further optimize the regimens for pneumonia, larger scale, multicenter prospective studies are needed.

## Conclusions

In conclusion, this study investigated the PK/PD relationship and the optimal dosage regimens of polymyxin B against CRO pneumonia in critically ill patients, and we found that AUC_ss,24 h_/MIC was the reliable predictive PK/PD index with the target of 66.9 for the treatment of nosocomial pneumonia caused by CRO in patients receiving combination therapy with another antimicrobial. Model-based simulation suggests 75 mg and 100 mg Q12 h maintenance dosage to achieve the comparable efficacy threshold. For patients unable to achieve the target concentration by intravenous administration, adjunctive inhalation of polymyxin B would be beneficial.

## Supplementary Information

**Additional file 1**. **Fig. S1a** Spearman’s rank correlation between peak, trough concentrations and AUC_ss_, _24_ _h_ (A) scatterplot of the peak plasma concentrations

**Additional file 2**. **Fig. S1b** Spearman’s rank correlation between peak, trough concentrations and AUC_ss,__24_ _h_ (B) scatterplot of the trough plasma concentrations. AUC_ss,__24_ _h_, the area under the plasma concentration-time curve across 24 hours at steady state

**Additional file 3**. **Table S1.** Univariate and Cox regression analysis of 30-day mortality

## Data Availability

The datasets generated during and/or analyzed during the current study are available from the corresponding author on reasonable request.

## Abbreviations

CRO: Carbapenem-resistant organism
HAP: Hospital acquired pneumonia
VAP: Ventilator-associated pneumonia
AKI: Acute kidney injury
PK: Pharmacokinetics
PD: Pharmacodynamics
PPK: Population pharmacokinetics
AUC_ss,24__ h_: Area under the concentration-time curve across 24 h at steady state
MIC: Minimum inhibitory concentration
*C*_min,ss_: Trough concentration at steady state
*C*_max,ss_: Peak concentration at steady state
CLSI: Clinical and laboratory standards institute
EUCAST: European Committee on Antimicrobial Susceptibility Testing
HAP: Hospital acquired pneumonia
CrCL: Creatinine clearance
CF: Clinical failure
CS: Clinical success
APACHE II: Acute Physiology and Chronic Health Enquiry
LLOD: Limit of quantification
FOCE-ELS: First-order conditional estimation-extended least square
OFV: Objective function value
SCM: Stepwise covariate modeling
pcVPC: Prediction-corrected visual predictive check
EBEs: Empirical Bayesian
ALT: Alanine aminotransferase
AST: Aspartate aminotransferase
TBIL: Total bilirubin
DBIL: Direct bilirubin
ALB: Serum albumin
CLd: Central compartment and peripheral compartment
Vp: Volume in peripheral compartment
IIV: Inter-individual variability
CL: Central compartment
Vc: Volume distribution in central compartment
IV: Intravenous
IH: Inhaled
PTA: Probability of target attainment
SD: Standard deviation
IQR: Interquartile range
HR: Hazard ratio
OR: Odds ratios
AOR: Adjusted odds ratios
ROC: Receiver operating characteristic
AUC_ROC_: Area under the diagnostic curve
