# Supplementary figures and images for: Optimization of polymyxin B regimens for the treatment of carbapenem-resistant organism nosocomial pneumonia: a real-world prospective study
Source: Crit Care. 2023 Apr 28;27:164. doi: 10.1186/s13054-023-04448-z (PMC10142183; doi:10.1186/s13054-023-04448-z)

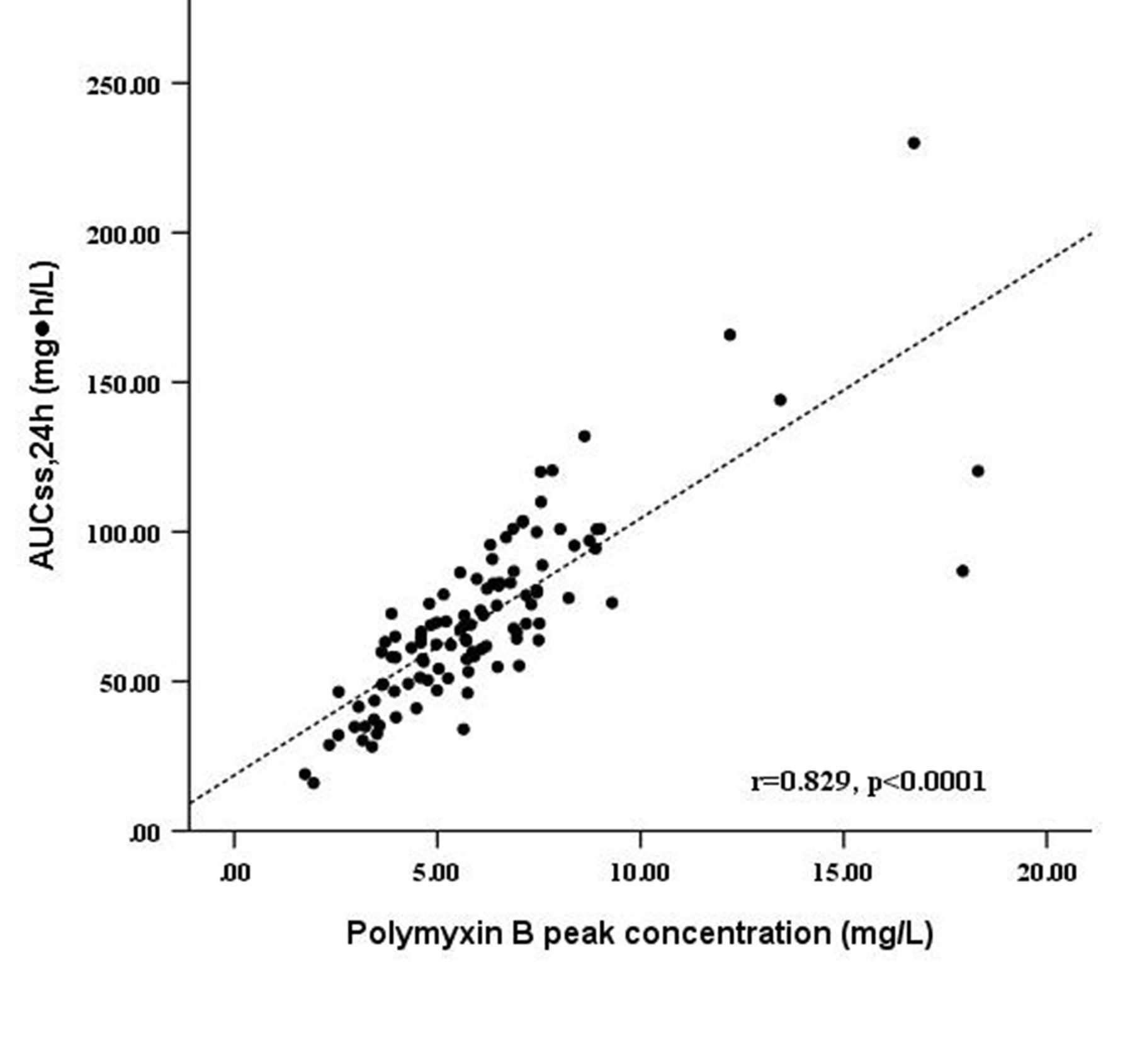

Supplement: Supplementary file 1 — Additional file 1. Fig. S1a Spearman’s rank correlation between peak, trough concentrations and AUCss, 24 h (A) scatterplot of the peak plasma concentrations [file 13054_2023_4448_MOESM1_ESM.jpeg]

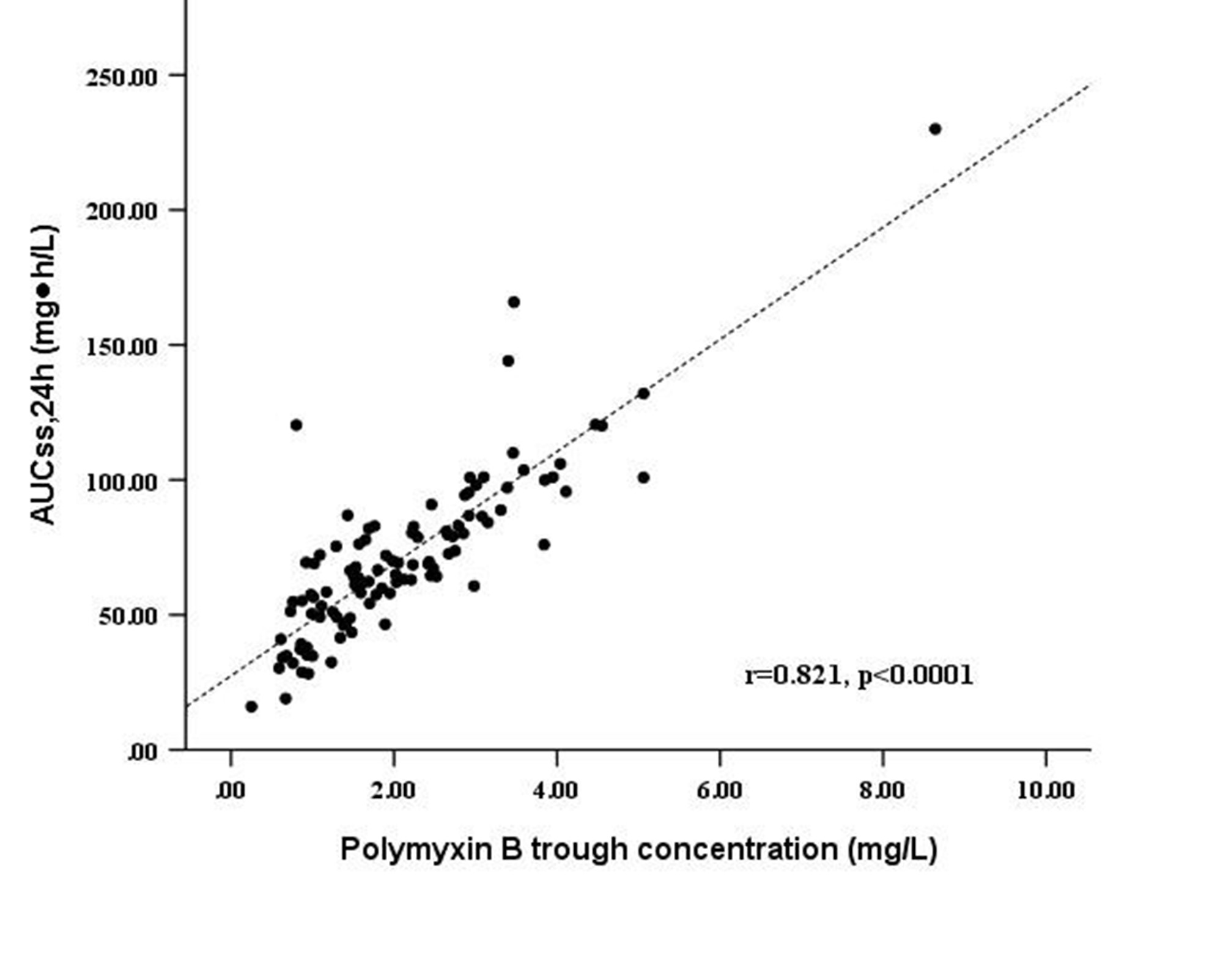

Supplement: Supplementary file 2 — Additional file 2. Fig. S1b Spearman’s rank correlation between peak, trough concentrations and AUCss, 24 h (B) scatterplot of the trough plasma concentrations. AUCss, 24 h, the area under the plasma concentration-time curve across 24 hours at steady state [file 13054_2023_4448_MOESM2_ESM.jpeg]
