# Supplementary material for: Optimization of polymyxin B regimens for the treatment of carbapenem-resistant organism nosocomial pneumonia: a real-world prospective study
Source: Crit Care. 2023 Apr 28;27:164. doi: 10.1186/s13054-023-04448-z (PMC10142183; doi:10.1186/s13054-023-04448-z)
Supplement: Supplementary file 3 — Additional file 3. Table S1. Univariate and Cox regression analysis of 30-day mortality [file 13054_2023_4448_MOESM3_ESM.docx]

**Additional file 1:**

**Fig. S1a** Spearman’s rank correlation between peak, trough concentrations and AUC_ss,24h_ (A) scatterplot of the peak plasma concentrations

**Additional file 2:**

**Fig. S1b** Spearman’s rank correlation between peak, trough concentrations and AUC_ss,24h_ (B) scatterplot of the trough plasma concentrations. AUC_ss,24h_, the area under the plasma concentration-time curve across 24 hours at steady state

**Additional file 3:**

**Table S1** Univariate and Cox regression model for 30-day mortality

| Variable | Survival^a^ (n = 77) | Non-survival^a^ (n = 28) | *p*^b^ | HR (95% CI) | *p*^c^ |
| --- | --- | --- | --- | --- | --- |
| Demographic parameters |  |  |  |  |  |
| Female | 20 (26.0%) | 12 (42.9%) | 0.079 |  |  |
| Age (years) | 64.0 (54.0, 76.0) | 67.5 (55.3, 76.0) | 0.534 |  |  |
| Weight (Kg) | 55.0 (50.0, 65.0) | 51.5 (50.0, 60.8) | 0.213 |  |  |
| Comorbidities |  |  |  |  |  |
| Sepsis | 25 (32.5%) | 16 (57.1%) | **0.017** |  |  |
| Pulmonary diseases | 12 (15.6%) | 3 (10.7%) | 0.298 |  |  |
| Heart disease | 46 (59.7%) | 12 (42.8%) | 0.184 |  |  |
| Diabetes mellitus | 12 (15.6%) | 7 (25%) | 0.236 |  |  |
| ARDS | 7 (9.10%) | 5 (17.8%) | 0.196 |  |  |
| Chronic liver disease | 21 (27.3%) | 8 (28.6%) | 0.812 |  |  |
| Chronic renal dysfunction | 15 (19.5%) | 10 (35.7%) | 0.071 |  |  |
| Solid tumor | 11 (14.3%) | 4 (14.3%) | 0.944 |  |  |
| Clinical conditions |  |  |  |  |  |
| Baseline CrCL (mL/min) | 83.6 (56.4, 118.3) | 60.9 (30.9, 84.1) | **0.018** |  |  |
| Albumin (g/L) | 30.8 (27.7, 33.7) | 30.4 (27.9, 35.6) | 0.407 |  |  |
| Baseline BUN (mmol/L) | 9.3 (6.3, 13.6) | 14.0 (8.2, 18.7) | 0.116 |  |  |
| APACHEII score | 17.0 (11.0, 21.0) | 24.5 (18.0, 28.0) | **0.004** | 1.07 (1.01-1.14) | 0.026 |
| Mechanical ventilation | 57 (74.0%) | 24 (85.7%) | 0.137 |  |  |
| Pathogens and susceptibility |  |  |  |  |  |
| CRAB | 62 (80.5%) | 24 (85.7%) | 0.357 |  |  |
| CRKP | 31 (40.3%) | 11 (39.3%) | 0.960 |  |  |
| CRPA | 16 (20.8%) | 7 (25.0%) | 0.347 |  |  |
| ≤ 0.5 mg/L | 2 (2.6%) | 1 (3.6%) | 0.453 |  |  |
| 1 mg/L | 75 (97.4%) | 27 (96.4%) | 0.453 |  |  |
| PMB treatment |  |  |  |  |  |
| Daily dose (mg) | 150.0 (100.0, 150.0) | 100.0 (100.0,150.0) | 0.272 |  |  |
| Daily dose/weight (mg/Kg) | 2.4 (2.0, 3.0) | 2.3 (2.0, 2.8) | 0.634 |  |  |
| Duration (day) | 12.5 (10.0, 16.0) | 9.0 (6.0, 13.2) | **0.003** | 0.67 (0.53-0.85) | 0.001 |
| Total dosage (mg) | 1625.0 (1200.0, 2400.0) | 1150.0 (637.5, 1637.5) | **0.008** |  |  |
| Total dosage (mg/Kg) | 30.0 (21.9, 40.6) | 20.8 (13.5, 25.2) | **0.009** |  |  |
| Combined with inhaled PMB | 26 (33.7%) | 8 (28.6%) | 0.700 |  |  |
| PMB concentration |  |  |  |  |  |
| AUC_ss, 24h_ (mg·h/L) | 69.4 (58.5, 86.9) | 62.1 (46.3, 83.2) | 0.126 |  |  |
| AUC_ss, 24h_/MIC | 69.7 (59.8, 110.0) | 62.1 (46.3, 90.9) | 0.245 |  |  |
| C_min,ss_ (μg/mL) | 2.2 (1.9, 2.5) | 2.1 (1.6, 2.6) | 0.658 |  |  |
| C_min,ss_ /MIC | 2.2 (1.9, 2.5) | 2.2 (1.6, 2.7) | 0.918 |  |  |
| C_max,ss_ (μg/mL) | 6.5 (5.8, 7.2) | 5.4 (4.6, 6.1) | 0.876 |  |  |
| C_max,ss_ /MIC | 6.6 (5.9,7.2) | 5.4 (4.6, 6.1) | 0.073 |  |  |
| Combination therapy |  |  |  |  |  |
| Carbapenems | 25 (32.5%) | 11 (39.3%) | 0.439 |  |  |
| Tigecycline | 25 (32.5%) | 8 (28.6%) | 0.791 |  |  |
| Ceftazidime avitabtam | 3 (3.9%) | 1 (3.6%) | 0.966 |  |  |
| Other β-lactam^d^ | 24 (32.2%) | 8 (28.6%) | 0.887 |  |  |
| Quinolone | 3 (3.9%) | 1 (3.6%) | 0.966 |  |  |
| Clinical response | 64 (83.1%) | 6 (21.4%) | **<0.0001** | 13.73 (4.21-44.76) | <0.0001 |
| Bacterial clearance | 35 (45.5%) | 9 (32.1%) | 0.284 |  |  |

*CI* Confidence interval, *OR* Odds ratio, *CrCL* Creatinine clearance, *APACHE* Acute physiology and chronic health evaluation, *BUN* Blood urea nitrogen, *PMB* Polymyxin B, *CRAB* Carbapenem-resistant *acinetobacter baumannii*, *CRKP* Carbapenem-resistant *klebsiella pneumonia*, *CRPA* Carbapenem-resistant *pseudomonas aeruginosa*, *AUC_ss,24h_* The area under the plasma concentration-time curve across 24 hours at steady state, *C_min,ss_* Steady-state trough plasma concentration, *C_max,ss_* Steady-state peak plasma concentration

^a^Categorical data are number (%) of subjects, continuous data are expressed as median (interquartile range, IQR).

^b^derived from univariate analysis

^c^derived from Cox regression analysis

^d^other β-lactam antibiotics include cefoperazone/sulbactam (*n* = 23) and piperacillin/tazobactam (*n* = 9)

Bold font indicates data with significant differences (*p* **<** 0.05)
